# Theoretical Analysis on Absorption of Carbon Dioxide (CO_2_) into Solutions of Phenyl Glycidyl Ether (PGE) Using Nonlinear Autoregressive Exogenous Neural Networks

**DOI:** 10.3390/molecules26196041

**Published:** 2021-10-05

**Authors:** Naveed Ahmad Khan, Muhammad Sulaiman, Carlos Andrés Tavera Romero, Fawaz Khaled Alarfaj

**Affiliations:** 1 Department of Mathematics, Abdul Wali Khan University Mardan, Mardan 23200, Khyber Pakhtunkhwa, Pakistan; ahmednaveed854477@gmail.com; 2 COMBA R&D Laboratory, Faculty of Engineering, Universidad Santiago de Cali, Cali 76001, Colombia; carlos.tavera00@usc.edu.co; 3 Department of Computer and Information Sciences, Imam Mohammad Ibn Saud Islamic University, Alhasa 31982, Saudi Arabia; fkarfaj@imamu.edu.sa

**Keywords:** carbon dioxide, phenyl glycidyl ether, reaction mechanisms, reaction kinetics and diffusion, chemical reactivity concentration of CO_2_ and PGE, artificial intelligence, machine learning, NARX networks

## Abstract

In this paper, we analyzed the mass transfer model with chemical reactions during the absorption of carbon dioxide (CO${}_{2}$) into phenyl glycidyl ether (PGE) solution. The mathematical model of the phenomenon is governed by a coupled nonlinear differential equation that corresponds to the reaction kinetics and diffusion. The system of differential equations is subjected to Dirichlet boundary conditions and a mixed set of Neumann and Dirichlet boundary conditions. Further, to calculate the concentration of CO${}_{2}$, PGE, and the flux in terms of reaction rate constants, we adopt the supervised learning strategy of a nonlinear autoregressive exogenous (NARX) neural network model with two activation functions (Log-sigmoid and Hyperbolic tangent). The reference data set for the possible outcomes of different scenarios based on variations in normalized parameters $\left(\alpha_{1},\;\alpha_{2},\;\beta_{1},\;\beta_{2},\;k\right)$ are obtained using the MATLAB solver “pdex4”. The dataset is further interpreted by the Levenberg–Marquardt (LM) backpropagation algorithm for validation, testing, and training. The results obtained by the NARX-LM algorithm are compared with the Adomian decomposition method and residual method. The rapid convergence of solutions, smooth implementation, computational complexity, absolute errors, and statistics of the mean square error further validate the design scheme’s worth and efficiency.

## 1. Introduction

Carbon dioxide is a generally useful gas made up of a carbon and two oxygen atoms. It is essential in plant photosynthesis, manufacturing carbonated soft drinks, powering pneumatic systems in robots, fire extinguishers, removing caffeine from coffee [1,2], etc. Carbon dioxide has the potential to be a significant and inexpensive carbon source. Its environmental impact as a greenhouse gas could be reduced by converting it into valuable products. The chemical conversion and fixation of carbon dioxide into valuable substances with desirable solutions has become an essential topic of research because of the danger posed by global warming. The conversion of carbon dioxide into useful chemicals is a very appealing approach [3].

Well-known examples include the oxirane reaction leading to a five-member cyclic carbonate [3], porous polymer bead-supported ionic liquids for the synthesis of cyclic carbonate [4,5], zeolite-based organic–inorganic hybrid catalysts for phosgene-free and solvent-free synthesis of cyclic carbonates, and carbamates [6,7]. Such carbonates are used as polymer synthesis sources and patented polar solvents.

The reaction kinetics of CO${}_{2}$ and phenyl glycidyl ether (PGE) was investigated by Park, and Choe [8] in a heterogeneous system during the chemical absorption of carbon dioxide into PGE solutions containing the catalyst THA–CP–MS41. The phenomena of absorption is modeled by nonlinear differential equations, which were solved by Wazwaz and Singh [9] to relate the steady-state concentration of CO${}_{2}$ and PGE using the homotopy analysis method.

S. Muthukaruppan [10] applied the Adomian decomposition method for the heat transfer of chemical reaction between CO${}_{2}$ and PGE solutions. Various numerical and analytical techniques, such as Laplace homotopy analysis method (LHAM) [11], homotopy perturbation transform method (HPTM) [12], optimal homotopy analysis method (OHAM) [13], conformable Adomian decomposition method (CADM) [14], and Haar wavelet method (HWM) [15], were developed to solve nonlinear differential equations governing reaction-diffusion equations of chemical kinetics.

In recent times, stochastic computing paradigms based on artificial intelligence have been used extensively to find numerical solutions for different problems arising in various fields, such as fuzzy systems [16,17,18], petroleum engineering [19], carbon capture process [20,21,22], wire coating dynamics [23], biological systems [24,25], civil engineering [26,27], coal-fired power plant retrofitted [28], and electrical and thermal engineering [29,30,31]. These contributions motivated the authors to investigate the absorption of carbon dioxide (CO${}_{2}$) into solutions of phenyl glycidyl ether (PGE) by strengthening the computational ability of neural networks. Salient features of the presented study are summarized as:

- A mathematical model for chemical analysis and absorption of carbon dioxide (CO${}_{2}$) into phenyl glycidyl ether (PGE) solutions is presented. Furthermore, a novel stochastic technique based on nonlinear autoregressive exogenous (NARX) neural networks with the Levenberg–Marquardt algorithm is utilized to optimize the system of singular nonlinear differential equations for the normalized concentration of CO${}_{2}$ and PGE.
- The design scheme NARX-LM algorithm with two different activations function (Log-sigmoid and Hyperbolic tangent) is implemented to investigate the influence of variations in normalized parameters such $\alpha_{1}$, $\alpha_{2}$, $\beta_{1}$ and $\beta_{2}$ on concentration profiles of CO${}_{2}$ and PGE.
- To validate the accuracy of the design supervised learning mechanism, the results obtained are compared with the residual method, the Adomian decomposition method, machine learning algorithms, and numerical solution.
- Extensive graphical analysis based on absolute errors, fitting of numerical and approximate solutions, absolute errors, and performance graphs of mean square error are plotted to further validate the worth of the design scheme.

## 2. Problem Formulation

**Figure 1:**
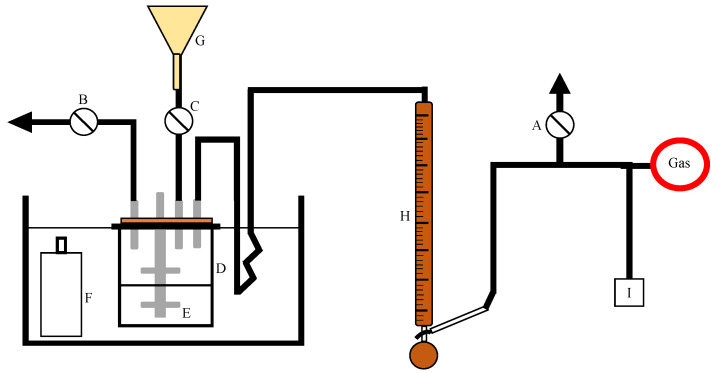
The schematic view of the stirred cell absorber.

**Figure 2:**
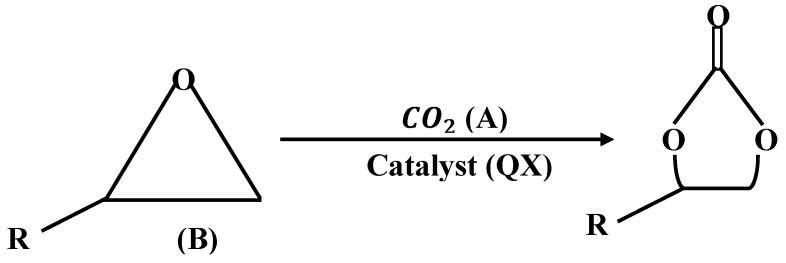
The overall reaction between CO${}_{2}$ and PGE.

Figure 1 demonstrates the experimental setup for stirred-cell absorber, where A, B, C are valves, D is the absorber, E is the impeller, F shows the bottle of liquid, G represents the funnel, H is the soap film meter, and I is the gas chromatographer [8]. The chemical reaction between carbon dioxide and phenyl glycidyl ether for the formation of a five membered cyclic carbonate is shown in Figure 2. Here, R is a functional group $\left(-{\mathrm{CH}}_{2}-O-C_{6}H_{5}\right)$. An overall reaction in Figure 2 consists of two steps, a reversible reaction between PGE(B) and THA-CP-MS41 (*QX*) is used to form an intermediate complex $C_{1}$ in the first step. In the second step, *QX* and five membered cyclic carbonate (*C*) is formed by a reversible reaction between CO${}_{2}$ and $C_{1}$.

(1) $$B+QX\overset{\xi_{1}}{\underset{\xi_{2}}{↔}}C_{1},$$

(2) $$A+C_{1}⟶\xi_{3}C+QX,$$

In a steady state, the chemical reaction rate of CO${}_{2}$ to $C_{1}$ is given as

(3) $$r_{A,cons}=\frac{S_{t}C_{B}}{\frac{1}{\Theta_{1}\xi_{3}C_{A}}+\frac{1}{\xi_{1}}+\frac{C_{B}}{\xi_{3}C_{A}}},$$

where $C_{A}$ and $C_{B}$ denotes the concentration of CO${}_{2}$ and PGE, $S_{t}$ is the surface area of the catalyst, and $\Theta_{1}$ denotes the reaction equilibrium. $\xi_{1}$ and $\xi_{2}$ denote the forward reaction constants in Equations (2) and (3), respectively. The nonlinear mass balances of CO${}_{2}$ and PGE as a result of the subsequent chemical reactions are shown in Equations (4) and (5) as

(4) $$D_{A}\frac{d^{2}C_{A}\left(z\right)}{dz^{2}}=\frac{S_{t}C_{B}\left(z\right)}{\frac{1}{\Theta_{1}\xi_{3}C_{A}\left(z\right)}+\frac{1}{\xi_{1}}+\frac{C_{B}\left(z\right)}{\xi_{3}C_{A}\left(z\right)}},$$

(5) $$D_{B}\frac{d^{2}C_{B}\left(z\right)}{dz^{2}}=\frac{S_{t}C_{B}\left(z\right)}{\frac{1}{\Theta_{1}\xi_{3}C_{A}\left(z\right)}+\frac{1}{\xi_{1}}+\frac{C_{B}\left(z\right)}{\xi_{3}C_{A}\left(z\right)}},$$

where $D_{A}$ and $D_{B}$ are parameters for the measure of diffusion of CO${}_{2}$ and PGE, respectively, *z* is distance. Boundary conditions for problem are

(6) $$\;\mathrm{at}\;\;z=0;\;C_{A}=C_{Ai},\;\frac{dC_{B}}{dz}=0,$$

(7) $$\;\mathrm{at}\;\;z=z_{L};\;C_{A}=C_{AL},\;C_{B}=C_{Bo},$$

The following dimensionless parameters are defined to normalize Equations (4) and (5) along with the boundary conditions.

(8) $$\begin{array}{c} u=\frac{C_{A}}{C_{Ai}},\;v=\frac{C_{B}}{C_{Bo}};\;x=\frac{z}{z_{L}},\;\alpha_{1}=\frac{z_{L}^{2}s_{t}C_{Bo}\Theta_{1}\xi_{3}}{D_{A}}\\ \alpha_{2}=\frac{z_{L}^{2}s_{t}C_{Ai}\Theta_{1}\xi_{3}}{D_{B}},\;\beta_{1}=\frac{C_{Ai}\Theta_{1}\xi_{3}}{\xi_{1}},\;\beta_{2}=\frac{C_{Bo}\Theta_{1}\xi_{1}}{\xi_{1}}, \end{array}$$

Now, using Equation (8), the nonlinear equations for diffusion of CO${}_{2}$ and PGE can be written as

(9) $$\frac{d^{2}u\left(x\right)}{dx^{2}}-\frac{\alpha_{1}u\left(x\right)v\left(x\right)}{1+\beta_{1}u\left(x\right)+\beta_{2}v\left(x\right)}=0,$$

(10) $$\frac{d^{2}\left(v\right)}{dx^{2}}-\frac{\alpha_{2}u\left(x\right)v\left(x\right)}{1+\beta_{1}u\left(x\right)+\beta_{2}v\left(x\right)}=0,$$

The dimensionless boundary conditions are given as

(11) $$\;\mathrm{At}\;\;x=0\;u=1,\;\frac{dv}{dx}=0,$$

(12) Atx=1u=k,v=1.

Here, the normalized concentrations of CO${}_{2}$ and PGE are denoted by u(x) and v(x), respectively. $\alpha_{1}$, $\alpha_{2}$, $\beta_{1}$, and $\beta_{2}$ are normalized parameters. The distance from the center is *x*, and $k=\frac{C_{AL}}{C_{Ai}}$ is the concentration of CO${}_{2}$ at the catalyst surface, and its value is less than 1. The enhancement factor of carbon dioxide and ratio of the flux (β) of the chemical reaction is defined as

(13) $$\beta =-{\left(\frac{du}{dx}\right)}_{x=0}.$$

## 3. Design Methodology

### 3.1. Artificial Neural Networks and NARX Model

Artificial Neural Networks (ANN’s) are used for an extensive range of problems in clustering, pattern classification, function approximation, recognition, optimization, and prediction [32,33]. ANNs are mathematical tools that are stimulated by the biological brain system, and they have a tremendous ability to learn, store, and remember data. They are black-box modelling tool that can perform non-linear mapping from an n-dimensional input space to an m-dimensional output space while the input and output spaces are unknown [34].

The choice of ANN model depends on the prior knowledge of the system to be modeled. The basic idea of NARX is a nonlinear version of the Autoregressive Exogenous (ARX) instrument, which is a common tool for identifying linear black-box systems. The NARX models are extensively used for modeling number of nonlinear dynamical system, such as dual response regulators interact with dual sensors [35], chaotic time series prediction [36], prediction of the daily direct solar radiation [37], and long-term time series prediction [38]. A recurrent dynamic neural network, or NARX, is a type of neural network that learns from previous experiences. It features feedback links that encircle the network in multiple levels.

**Figure 3:**
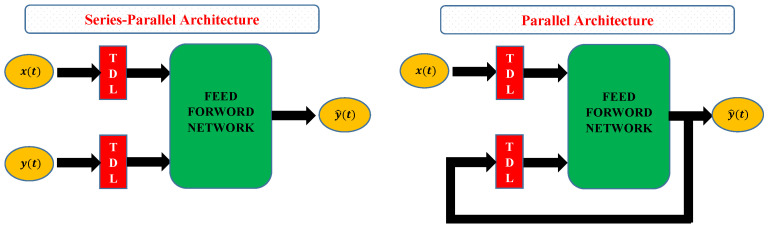
Architectures of the NARX neural network.

NARX has two different architectures named series-parallel architecture (open-loop) and parallel architecture (close-loop) as shown in Figure 3. In this study, a parallel architecture NARX model is adopted to study the concentration and absorption of CO${}_{2}$ into a PGE solution. The general NARX model is given as

(14) $$\hat{y}\left(t+1\right)=F\left(\begin{array}{c} y\left(t\right),y\left(t-1\right),\ldots ,y\left(t-n_{y}\right),x\left(t+1\right)\\ x\left(t\right),x\left(t-1\right),\ldots ,x\left(t-n_{x}\right) \end{array}\right)$$

Here, *t* represents the time period, $\hat{y}\left(t+1\right)$ is output of the NARX at time *t*, and $n_{x}$ and $n_{y}$ are the input and output delays. F(.) is the mapping function of the neural networks. The basic advantage of using parallel architecture is that the usual training algorithm for Multi-Layer Perceptron (MLP) can be used for training neurons. The MLP offers a powerful structure that allows learning any type of continuous nonlinear mapping. A traditional MLP has three layers: input, hidden, and output. Neurons, activation functions, and weights are the other components.

In this study, we used two activation functions named the Log-sigmoid and Hyperbolic tangent. The convergence speed of these functions is much higher then other activation functions. The optimal gradient factors for Logsigmoid and Hyperbolic tangent are greater than those for Normal, Cauchy, Erf-Logsig and Laplace activations functions, which make them unique. The mathematical form for these activation functions are given by Equations (15) and (16), respectively.

(15) $$f_{1}\left(x\right)=\frac{1}{1+e^{-x}},$$

(16) $$f_{2}\left(x\right)=\frac{e^{x}-e^{-x}}{e^{x}+e^{-x}},$$

**Figure 4:**
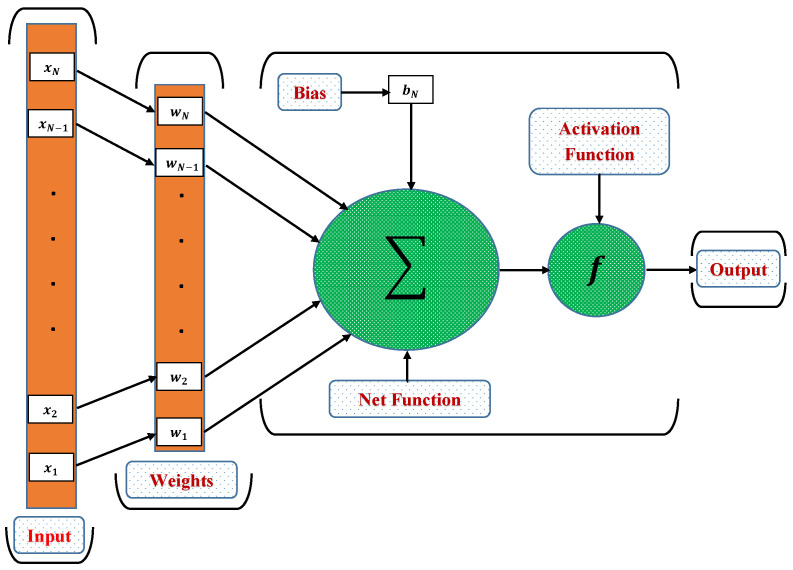
Details of a neuron in an MLP network.

The detailed structure of neurons along with different layers of MLP network are shown in Figure 4. The motivation of using NARX model with respect to other neural networks model is its speed of convergence and needs of less training cycles [39]. It provides the description of the system in terms of nonlinear function of delayed inputs, outputs, and their predicted errors. Thus, NARX model generalizes any nonlinear dynamical system and can be applied to various problems of different fields, such as nonlinear filtering, prediction, chaotic time series prediction, control, and time series modeling [40].

### 3.2. Learning Procedure and Performance Indicators

In this section, the working and training procedure of neurons is discussed. An appropriate algorithm is used to train the weights for calculating an approximate solution for the problem. During the training phase, a network is presented with a set of inputs and their desired output (also known as target data). A reference solution or target data of 1001 points is generated by using the numerical solver “Pdex4” in MATLAB. Furthermore, the data and weights are tuned by backpropogated Levenberg–Marquardt algorithm using "nntool" for proper training, validation, and testing. The sample of 1001 points is divided as

- 75% (701 samples) are used for training.
- 15% (150 samples) are used for validation.
- 15% (150 samples) are used for testing.

**Figure 5:**
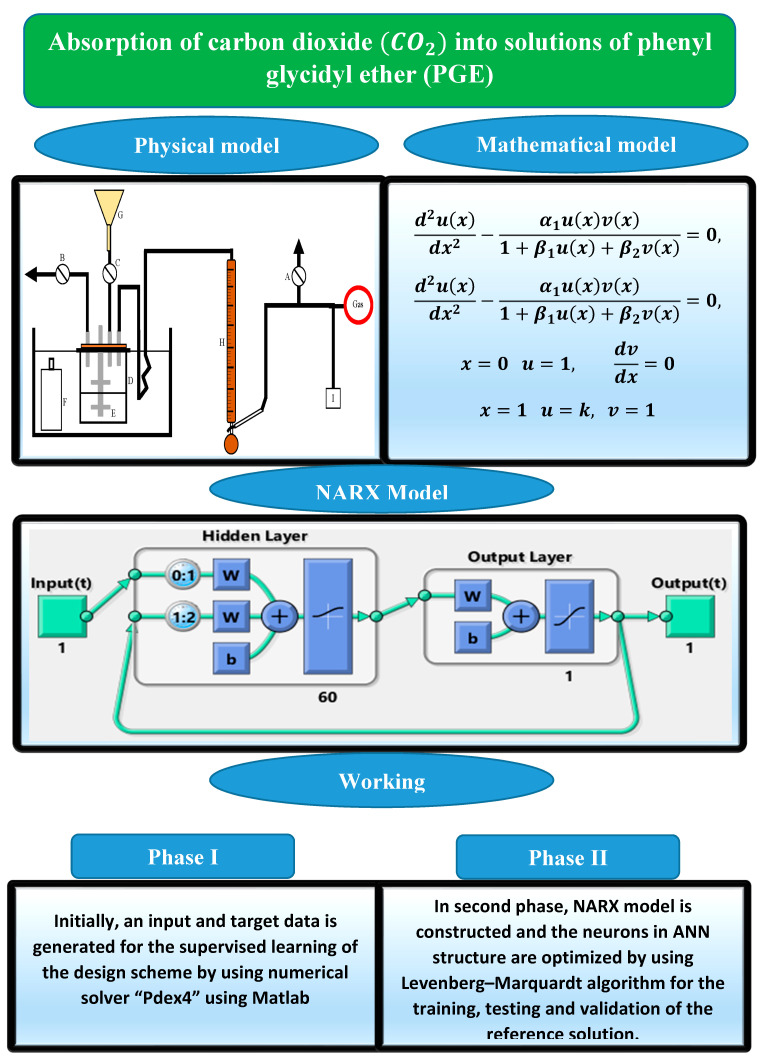
Mathematical model of the problem, NARX model, and workflow of the design scheme.

Figure 5 shows the model of the problem, NARX model, and workflow of the design scheme.

The performance of design scheme are measures of the performance indicators in terms of the mean square error (MSE) of the fitness function of the model, regression $R^{2}$, error histograms, and absolute errors (AE). The mathematical formulation of the MSE, $R^{2}$, and AE are given as

(17) $$\mathrm{MSE}=\frac{1}{m}\sum_{j=1}^{m}{\left(x_{j}\left(t\right)-{\hat{x}}_{j}\left(t\right)\right)}^{2},$$

(18) $$R^{2}=1-\frac{\sum_{j=1}^{m}{\left({\hat{x}}_{j}\left(t\right)-{\bar{x}}_{j}\left(t\right)\right)}^{2}}{\sum_{j=1}^{m}{\left(x_{j}\left(t\right)-{\bar{x}}_{j}\left(t\right)\right)}^{2}},$$

and

(19) $$\mathrm{AE}=\left|x_{j}\left(t\right)-{\hat{x}}_{j}\left(t\right)\right|,\;j=1,2,\ldots ,m.$$

where, $x_{j}$, ${\bar{x}}_{j}$, and ${\hat{x}}_{j}$ denote the reference, approximate, and mean of the solution at the jth input, and *m* is the number of mesh points. The desire value of MSE and AE for perfect fitting is equal to zero, while the value of $R^{2}$ is one.

## 4. Reference Solutions

In the literature, various methods have been developed to study the concentration of carbon dioxide, phenyl glycidyl ether, and enhancement factors.

Approximate solutions obtained by the the Adomian decomposition method and Duan–Rach modified (ADM and DRM) [41] are

(20) $$\begin{array}{cc} \; & u\left(x\right)=1-\left[\frac{\left.x\left(1-x\right)\alpha_{1}+2\left(1-k\right)(1+\beta_{1}+\beta_{2}\right)}{2\left(1+\beta_{1}+\beta_{2}\right)}\right]+\frac{xa_{1}}{24{\left(1+\beta_{1}+\beta_{2}\right)}^{3}}\\ \; & \left[5\alpha_{2}\left(1+\beta_{1}\right)+a_{1}\left(1+\beta_{2}\right)+4\left(1-k\right)\left(1+\beta_{2}\right)\left(1+\beta_{1}+\beta_{2}\right)-x\left(\begin{array}{c} \left(6-x^{2}\right)\alpha_{2}\left(1+\beta_{1}\right)+\left(2-x\right)xa_{1}\left(1+\beta_{2}\right)\\ +4\left(1-k\right)x\left(1+\beta_{2}\right)\left(1+\beta_{1}+\beta_{2}\right) \end{array}\right)\right] \end{array}$$

(21) $$\begin{array}{cc} \; & v\left(x\right)=1-\frac{\left(1-x^{2}\right)\alpha_{2}}{2\left(1+\beta_{1}+\beta_{2}\right)}+\frac{\alpha_{2}}{24{\left(1+\beta_{1}+\beta_{2}\right)}^{3}}\\ \; & \left[5\alpha_{2}\left(1+\beta_{1}\right)+\alpha_{1}\left(1+\beta_{2}\right)+4\left(1-k\right)\left(1+\beta_{2}\right)\left(1+\beta_{1}+\beta_{2}\right)-x^{2}\left(\begin{array}{c} \left(6-x^{2}\right)\alpha_{2}\left(1+\beta_{1}\right)+\left(2-x\right)x\alpha_{1}\left(1+\beta_{2}\right)\\ +4\left(1-k\right)x\left(1+\beta_{2}\right)\left(1+\beta_{1}+\beta_{2}\right) \end{array}\right)\right] \end{array}$$

Approximate solutions obtained by the domian decomposition method [10] are

(22) $$\begin{array}{cc} \; & u\left(x\right)=\left(k-1\right)x+1+\frac{\alpha_{1}x}{2\beta_{1}}\left(x-1\right)-\frac{\alpha_{1}\left(1+\beta_{2}\right)}{\beta_{1}^{3}{\left(k-1\right)}^{2}}\left[\log\left(1+\beta_{2}+\beta_{1}\right)-1\right]\left(1+\beta_{2}+\beta_{1}\right)\left(x-1\right)\\ \; & \frac{\alpha_{1}\left(1+\beta_{2}\right)}{\beta_{1}^{3}{\left(k-1\right)}^{2}}\left[\begin{array}{c} \left(\log\left(1+\beta_{2}+\beta_{1}\left(\left(k-1\right)x+1\right)-1\right)\right)\left(\log\left(1+\beta_{2}+\beta_{1}\left(\left(k-1\right)x+1\right)-1\right)\right)\\ -x\left(\log\left(1+\beta_{2}+\beta_{1}k\right)-1\right)\left(1+\beta_{2}+\beta_{1}k\right) \end{array}\right], \end{array}$$

(23) $$\begin{array}{cc} \; & v\left(x\right)=1+\frac{\alpha_{2}}{2\beta_{1}}\left(x^{2}-1\right)+\frac{\alpha_{2}\left(1+\beta_{2}\right)\left(x-1\right)}{\beta_{1}^{2}\left(k-1\right)}\left[\log\left(1+\beta_{2}+\beta_{1}\right)\right]\\ \; & -\frac{\alpha_{2}\left(1+\beta_{2}\right)}{\beta_{1}^{3}{\left(k-1\right)}^{2}}\left[\begin{array}{c} \left.\left(\log\left(1+\beta_{2}+\beta_{1}\left(\left(k-1\right)x+1\right)-1\right)\right)\left(1+\beta_{2}+\beta_{1}\left(\left(k-1\right)x+1\right)-1\right)\right)\\ -\left(\log\left(1+\beta_{2}+\beta_{1}k\right)-1\right)\left(1+\beta_{2}+\beta_{1}k\right) \end{array}\right] \end{array}$$

Approximate solutions obtained by the Adomian Daftarder–Jafari method [42] are

(24) $$u\left(x\right)=x+\frac{x^{4}\alpha_{1}}{12}+\frac{x^{7}\alpha_{1}^{2}}{504}+\frac{x^{7}\alpha_{1}\alpha_{2}}{504}+\frac{x^{10}\alpha_{1}^{2}\alpha_{2}}{12960}-\frac{x^{5}a_{1}\beta_{1}}{20}-\frac{x^{8}\alpha_{1}^{2}\beta_{1}}{672}-\frac{x^{5}a_{1}\beta_{2}}{20}-\frac{x^{8}\alpha_{1}\alpha_{2}\beta_{2}}{672},$$

(25) $$v\left(x\right)=x+\frac{x^{4}\alpha_{2}}{12}+\frac{x^{7}\alpha_{1}\alpha_{2}}{504}+\frac{x^{7}\alpha_{2}^{2}}{504}+\frac{x^{10}\alpha_{2}^{2}\alpha_{1}}{12960}-\frac{x^{5}a_{2}\beta_{1}}{20}-\frac{x^{8}a_{1}a_{2}\beta_{1}}{672}-\frac{x^{5}a_{2}\beta_{2}}{20}-\frac{x^{8}\alpha_{2}^{2}\beta_{2}}{672},$$

The approximate solutions obtained by the Residual method [43] are

(26) $$u\left(x\right)=k\frac{\sinh\left(\sqrt{\frac{\alpha_{1}}{1+\beta_{1}k+\beta_{2}}}x\right)}{\sinh\left(\sqrt{\frac{\alpha_{1}}{1+\beta_{1}k+\beta_{2}}}\right)}+\frac{\sinh\left(\sqrt{\frac{\alpha_{1}}{1+\beta_{1}k+\beta_{2}}}\left(1-x\right)\right)}{\sinh\left(\sqrt{\frac{\alpha_{1}}{1+\beta_{1}k+\beta_{2}}}\right)}$$

(27) $$v\left(x\right)=\frac{\cosh\left(\sqrt{\frac{\alpha_{2}k}{1+\beta_{1}k+\beta_{2}}}x\right)}{\cosh\left(\sqrt{\frac{\alpha_{2}k}{1+\beta_{1}k+\beta_{2}}}\right)},$$

## 5. Numerical Experimentation and Discussion

**Figure 6:**
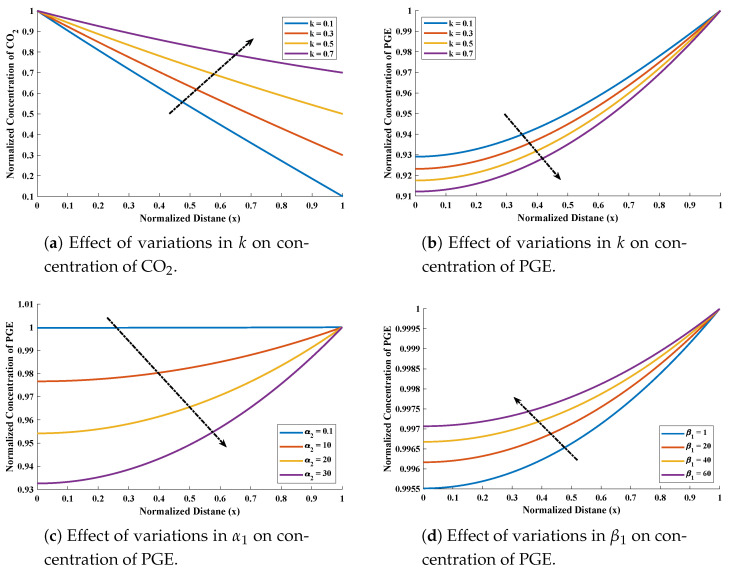

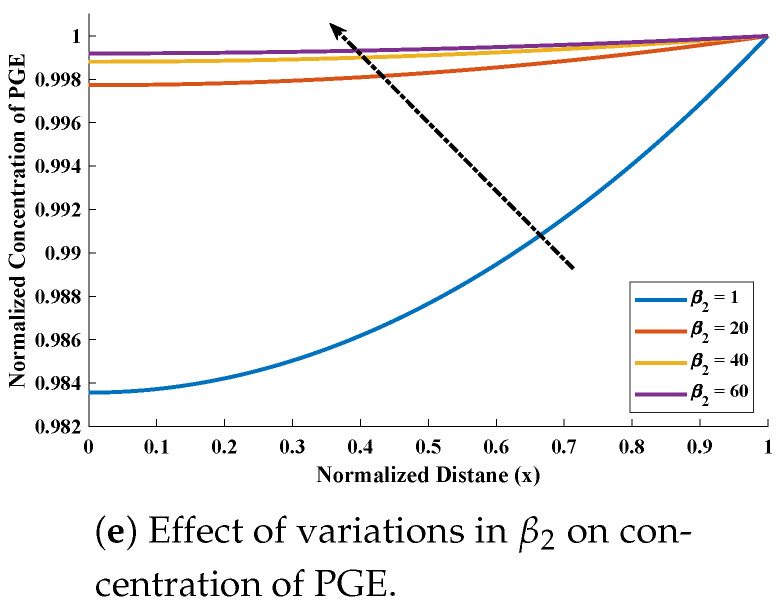
Influence of variations in different parameters on the normalized concentration of CO${}_{2}$ and PGE.

In this section, the design scheme NARX-LM algorithm is applied to study the concentration of CO${}_{2}$ and PGE solution under influence of variations in normalized parameters. Figure 6a,b represents the effect of variations in *k* on CO${}_{2}$ and PGE with $\alpha_{1}=\alpha_{2}=\beta_{1}=1$ and $\beta_{2}=3$. Figure 6c illustrates the influence of variations in $\alpha_{2}$ with $\alpha_{1},\;\beta_{1}=100,\;k=0.1$ and $\beta_{2}=10$. Variations in $\beta_{1}$ and $\beta_{2}$ with fixed values of $\alpha_{1}=\alpha_{2}=1$ and k=0.1 are shown through Figure 6d,e, respectively. It can be seen that concentration of CO${}_{2}$ increases with increase in *k*.

**Figure 7:**
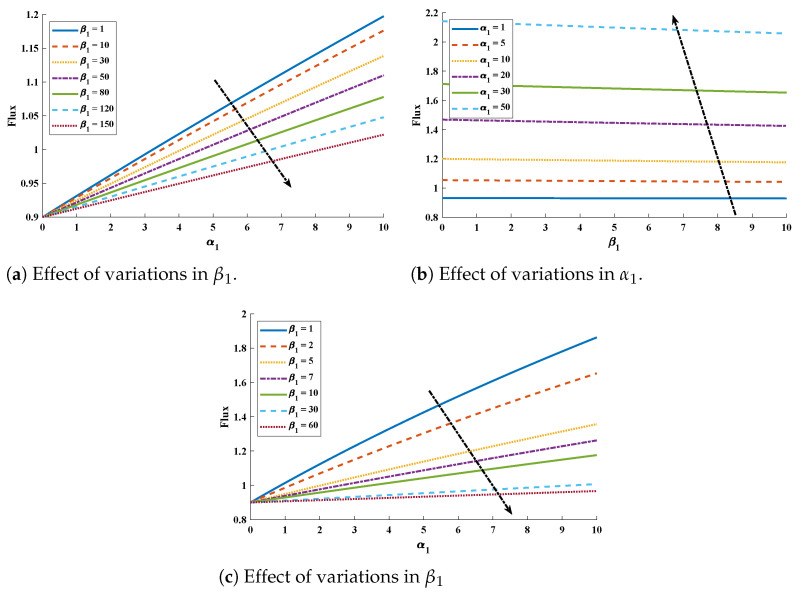
Influence of variations on the normalized parameter flux (enhancement factor).

The diffusivity of PGE decreases with increases in the surface catalyst. Influence of variations in flux (Enhancement factor) was investigated and the results are demonstrated in Figure 7. The value of flux decreases with increase in $\beta_{1}$ and $\beta_{2}$ while it increase with increase in $\alpha_{1}$. Further, to study, the results of design scheme different cases of Equations (9) and (10) are considered. Csse I: $k=0.1,\;\beta_{1}=0.1,\;\beta_{2}=0.001,\;\alpha_{1}=1$ and $\alpha_{2}=1$, Case II: $k=0.5,\;\beta_{1}=1,\;\beta_{2}=3,\;\alpha_{1}=2$ and $\alpha_{2}=2$, Case III: $k=0.1,\;\beta_{1}=100,\;\beta_{2}=10,\;\alpha_{1}=1$ and $\alpha_{2}=50$ and Case IV: $k=0.5,\;\beta_{1}=1,\;\beta_{2}=3,\;\alpha_{1}=2$, and $\alpha_{2}=5$.

The statistics of approximate solutions obtained by NARX-NM algorithm for steady state concentration profiles of CO${}_{2}$ and PGE are compared with the Adomian decomposition method and Duan–Rach modified (ADM and DRM) [41], Adomian decomposition method (ADM) [10], Residual method [43], and numerical method as shown in Table 1 and Table 2. The results in terms of absolute errors obtained by NARX-LM algorithm are compared with machine learning techniques, such as feed-forward backpropogated (FF) and Layer-Recurrent (LR) neural networks. Table 3 and Table 4 shows that solutions obtained by proposed technique are in good agreement with analytical solutions as compared to other neural networks.

**Figure 8:**
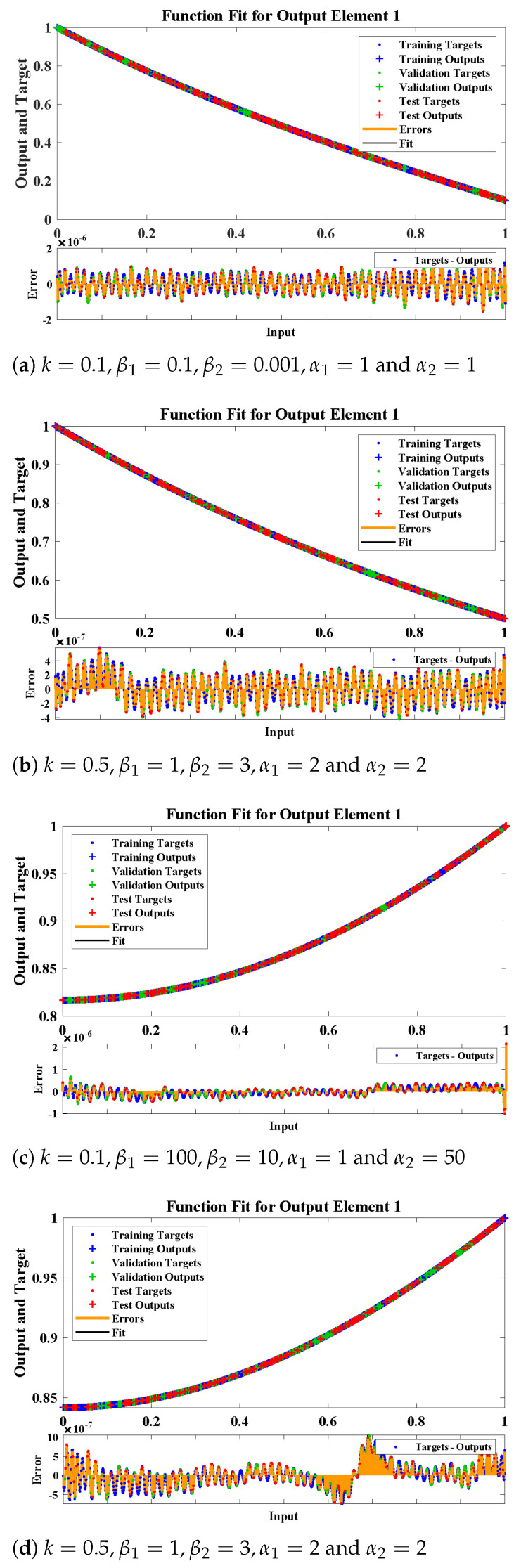
(**a**,**b**) The fitting of numerical solution with the results obtained by the NARX-LM algorithm for the concentration of CO${}_{2}$ and (**c**,**d**) the concentration of PGE.

**Figure 9:**
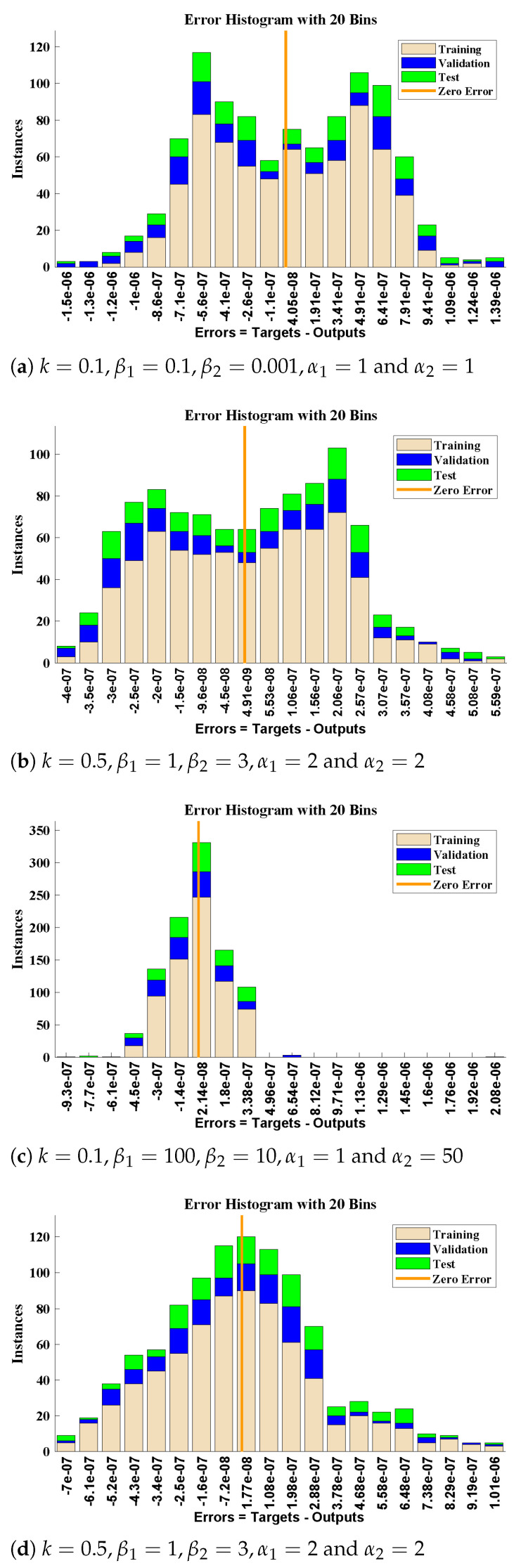
(**a**,**b**) The error histogram analysis for the concentration of CO${}_{2}$ and (**c**,**d**) the errors in solution for the concentration of PGE.

**Figure 10:**
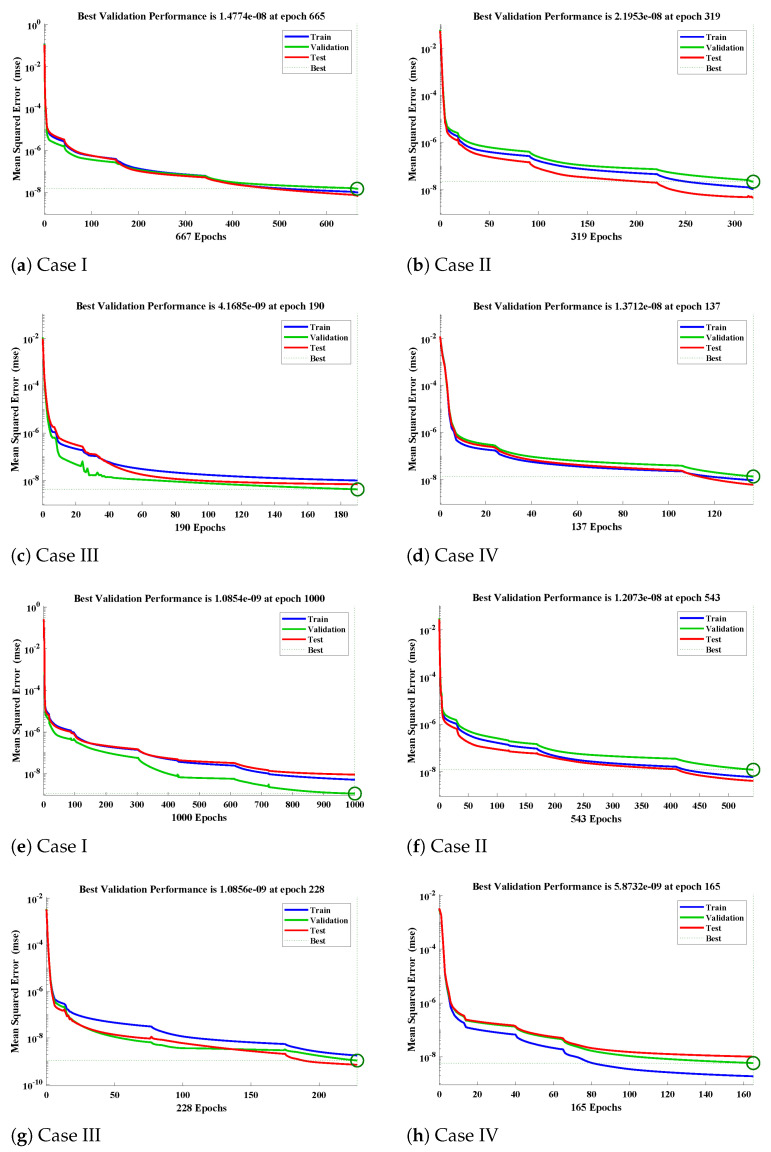
(**a**–**d**) The convergence value of the performance function using the hyperbolic tangent sigmoid function and (**e**–**h**) the results of the performance function with the Log-sigmoid activation function.

The fitting of approximate solutions by the design algorithm with reference data for different cases of Equations (9) and (10) are shown in Figure 8. The absolute errors (AE) in our solutions are shown through Figure 8 and Figure 9. It can be seen that solutions by NARX-LM algorithm overlaps the numerical solutions with AE that lies around $10^{-6}$ to $10^{-8}$, $10^{-7}$ to $10^{-9}$, $10^{-6}$ to $10^{-8}$ and $10^{-7}$ to $10^{-8}$, respectively. The performance of the design scheme for two activation functions in term of mean square error are shown in Figure 10.

The values of the mean square error obtained with the hyperbolic tangent sigmoid function for different cases are $1.4774\times 10^{-08}$, $2.1953\times 10^{-08}$, $4.1685\times 10^{-09}$ and $1.3712\times 10^{-08}$ with gradient $5.7587\times 10^{-08}$, $9.2706\times 10^{-08}$, $9.1040\times 10^{-08}$ and $8.1414\times 10^{-08}$, respectively. The statistics of the performance function with the Log-sigmoid activation function for different cases are $1.0854\times 10^{-09}$, $1.2073\times 10^{-08}$, $1.0856\times 10^{-09}$ and $5.8732\times 10^{-09}$ with gradient $5.6208\times 10^{-07}$, $2.2750\times 10^{-09}$, $9.7326\times 10^{-08}$, and $9.9145\times 10^{-08}$, respectively.

## 6. Conclusions

In this paper, we examined the system of nonlinear differential equations that relates the steady state concentration of carbon dioxide and phenyl glycidyl ether. To study the chemical analysis and absorption of carbon dioxide (CO${}_{2}$) into phenyl glycidyl ether (PGE) solutions, a novel stochastic technique based on nonlinear autoregressive exogenous (NARX) neural networks with the Levenberg–Marquardt algorithm was designed. The design scheme NARX-LM algorithm with two different activations function (Log-sigmoid and Hyperbolic tangent) was implemented to investigate the influence of variations in normalized parameters, such as $\alpha_{1},\;\alpha_{2},\;\beta_{1}$, and $\beta_{2}$ on concentration profiles of CO${}_{2}$ and PGE.

Extensive graphical and statistical analysis illustrated that increases in *k* increased the concentration of CO${}_{2}$. The diffusivity of PGE decreased with increases in $\beta_{1}$ and $\beta_{2}$. The approximate solutions obtained by the NARX-LM algorithm were compared with state-of-the-art techniques. Statistics dictates that the designs scheme overlapped the numerical solutions with minimum absolute errors. Convergence graphs and error histograms analysis further validated the worth of the design scheme.

## Figures and Tables

**Table 1:** Comparison of the solutions obtained by the NARX-LM algorithm with the numerical results, NM, ADM, and DRM for the steady state concentration of CO${}_{2}$.

	$k=0.1,\;\beta_{1}=0.1,\;\beta_{2}=0.001,\;\alpha_{1}=1$ and $\alpha_{2}=1$					$k=0.5,\;\beta_{1}=1,\;\beta_{2}=3,\;\alpha_{1}=2$ and $\alpha_{2}=2$				
x	ADM & DRM	ADM	RM	Numerical	NARX-LM	ADM & DRM	ADM	RM	Numerical	NARX-LM
0	1	1	1	1	**1**	1	1	1	1	**1**
0.2	0.7721	0.7722	0.7733	0.7754	**0.7754**	8734	8734	8730	8740	**8740**
0.4	0.5746	0.5746	0.5773	0.5797	**0.5797**	7614	7614	7610	7620	**7620**
0.6	0.401	0.4011	0.4042	0.4061	**0.4061**	6629	6629	6626	6629	**6629**
0.8	0.2451	0.2451	0.2472	0.2482	**0.2482**	5762	5762	5761	5758	**5758**
1.0	0.1	0.1	0.1	0.1	**0.1**	0.5	0.5	0.5	0.5	**0.5**

**Table 2:** Comparison of the solutions obtained by the NARX-LM algorithm with the numerical results, NM, ADM, and DRM for the steady state concentration of PGE.

	$k=0.1,\;\beta_{1}=100,\;\beta_{2}=10,\;\alpha_{1}=1$ and $\alpha_{2}=50$					$k=0.5,\;\beta_{1}=1,\;\beta_{2}=3,\;\alpha_{1}=2$ and $\alpha_{2}=2$				
x	ADM & DRM	ADM	RM	Numerical	NARX-LM	ADM & DRM	ADM	RM	Numerical	NARX-LM
0	0.8199	0.8098	0.8199	0.8173	**0.8173**	0.8426	0.842	0.8426	0.842	**0.842**
0.2	0.827	0.8188	0.827	0.826	**0.826**	0.8497	0.8491	0.8497	0.8489	**0.8489**
0.4	0.8483	0.8459	0.8483	0.8478	**0.8478**	0.8701	0.8698	0.8701	0.8688	**0.8688**
0.6	0.8839	0.881	0.8839	0.8849	**0.8849**	0.9026	0.9021	0.9026	0.9023	**0.9023**
0.8	0.9342	0.9331	0.9342	0.9345	**0.9345**	0.9462	0.9459	0.9462	0.9471	**0.9471**
1.0	1	1	1	1	**1**	1	1	1	1	**1**

**Table 3:** Comparison of the absolute errors in solutions for the concentration of CO${}_{2}$ obtained by the NARX-LM algorithm with different machine learning techniques for Case I and II.

	Case I			Case II		
*x*	FF-NN	LR-NN	NARX-LM	FF-NN	LR-NN	NARX-LM
0.0	7.567414 $\times 10^{-04}$	1.976408$\times 10^{-03}$	**3.664701** $\times 10^{-04}$	1.853876$\times 10^{-03}$	1.611739$\times 10^{-03}$	**1.594322** $\times 10^{-04}$
0.1	2.294808$\times 10^{-05}$	3.465679$\times 10^{-05}$	**2.856463** $\times 10^{-06}$	4.781680$\times 10^{-05}$	2.421558$\times 10^{-05}$	**1.214353** $\times 10^{-06}$
0.2	**8.805666$\times 10^{-07}$**	3.535741$\times 10^{-05}$	1.233261$\times 10^{-06}$	8.706504$\times 10^{-06}$	3.312607$\times 10^{-05}$	**3.532873$\times 10^{-08}$**
0.3	5.146110$\times 10^{-06}$	2.495271$\times 10^{-05}$	**8.119822$\times 10^{-07}$**	1.454172$\times 10^{-05}$	1.755399$\times 10^{-05}$	**4.475227$\times 10^{-07}$**
0.4	2.780715$\times 10^{-06}$	2.560681$\times 10^{-05}$	**7.753534$\times 10^{-07}$**	4.615430$\times 10^{-06}$	1.804920$\times 10^{-05}$	**2.842042$\times 10^{-07}$**
0.5	1.359414$\times 10^{-06}$	2.127227$\times 10^{-05}$	**5.663649$\times 10^{-07}$**	4.426592$\times 10^{-06}$	1.888973$\times 10^{-05}$	**2.552623$\times 10^{-07}$**
0.6	9.394572$\times 10^{-07}$	1.570394$\times 10^{-05}$	**5.437249$\times 10^{-07}$**	9.599460$\times 10^{-06}$	1.659002$\times 10^{-05}$	**1.624006$\times 10^{-07}$**
0.7	4.026649$\times 10^{-07}$	9.583692$\times 10^{-06}$	**3.555038$\times 10^{-08}$**	8.586999$\times 10^{-06}$	1.037132$\times 10^{-05}$	**1.193209$\times 10^{-07}$**
0.8	9.554468$\times 10^{-07}$	1.991985$\times 10^{-05}$	**1.297162$\times 10^{-08}$**	1.651644$\times 10^{-05}$	1.402911$\times 10^{-05}$	**3.752629$\times 10^{-07}$**
0.9	6.361145$\times 10^{-07}$	5.625482$\times 10^{-05}$	**1.278474$\times 10^{-07}$**	4.498823$\times 10^{-05}$	7.522469$\times 10^{-05}$	**9.783728$\times 10^{-07}$**
1.0	1.073576$\times 10^{-03}$	1.483420$\times 10^{-03}$	**3.995772$\times 10^{-04}$**	9.093789$\times 10^{-04}$	1.069798$\times 10^{-03}$	**2.023171$\times 10^{-04}$**

**Table 4:** Comparison of the absolute errors in solutions for the concentration of PGE obtained by the NARX-LM algorithm with different machine learning techniques for Case III and IV.

	Case III			Case IV		
*x*	FF-NN	LR-NN	NARX-LM	FF-NN	LR-NN	NARX-LM
0.0	5.662392$\times 10^{-05}$	1.282375$\times 10^{-04}$	**2.454875$\times 10^{-06}$**	9.311650$\times 10^{-05}$	2.660996$\times 10^{-04}$	**8.705075$\times 10^{-07}$**
0.1	7.540143$\times 10^{-06}$	3.653438$\times 10^{-05}$	**1.241626$\times 10^{-07}$**	5.827090$\times 10^{-05}$	9.107916$\times 10^{-05}$	**8.132946$\times 10^{-08}$**
0.2	2.002106$\times 10^{-05}$	2.879628$\times 10^{-05}$	**5.167114$\times 10^{-09}$**	3.032041$\times 10^{-05}$	1.850514$\times 10^{-04}$	**6.939629$\times 10^{-09}$**
0.3	3.071841$\times 10^{-05}$	4.926253$\times 10^{-05}$	**9.863673$\times 10^{-08}$**	5.746880$\times 10^{-05}$	3.007176$\times 10^{-05}$	**5.252565$\times 10^{-08}$**
0.4	1.557625$\times 10^{-05}$	5.678588$\times 10^{-05}$	**6.458083$\times 10^{-08}$**	4.507465$\times 10^{-05}$	1.119539$\times 10^{-04}$	**5.121962$\times 10^{-08}$**
0.5	2.451808$\times 10^{-05}$	4.759676$\times 10^{-05}$	**7.378733$\times 10^{-08}$**	1.396936$\times 10^{-05}$	6.004553$\times 10^{-05}$	**4.057286$\times 10^{-08}$**
0.6	7.209635$\times 10^{-06}$	2.690070$\times 10^{-05}$	**6.402087$\times 10^{-08}$**	5.687769$\times 10^{-05}$	1.139300$\times 10^{-04}$	**7.379871$\times 10^{-08}$**
0.7	2.222096$\times 10^{-05}$	1.090769$\times 10^{-04}$	**4.285971$\times 10^{-08}$**	5.169435$\times 10^{-05}$	1.277712$\times 10^{-04}$	**2.756274$\times 10^{-08}$**
0.8	1.854701$\times 10^{-05}$	2.853953$\times 10^{-05}$	**1.863085$\times 10^{-07}$**	7.419310$\times 10^{-07}$	3.113386$\times 10^{-05}$	**5.949529$\times 10^{-09}$**
0.9	**4.618550$\times 10^{-09}$**	1.901168$\times 10^{-04}$	5.167832$\times 10^{-07}$	3.146698$\times 10^{-05}$	1.796623$\times 10^{-04}$	**3.940062$\times 10^{-08}$**
1.0	1.150652$\times 10^{-03}$	1.836329$\times 10^{-03}$	**7.934356$\times 10^{-05}$**	1.193031$\times 10^{-03}$	2.681365$\times 10^{-03}$	**2.036388$\times 10^{-04}$**

## Data Availability

The data that support the findings of this study are available from the corresponding author upon reasonable request.
